# Vaping-Associated Pneumomediastinum: A Case Report

**DOI:** 10.7759/cureus.110696

**Published:** 2026-06-11

**Authors:** Markus D Moore, Margarita Taburyanskaya, Adil K Shivji, Saeed K Alzghari

**Affiliations:** 1 Pharmacy, Texas Health Harris Methodist Hospital Fort Worth, Fort Worth, USA

**Keywords:** e-cigarettes, pneumomediastinum, subcutaneous emphysema, vaping, vaping-associated pneumomediastinum

## Abstract

Despite known health risks, the use of e-cigarettes among adolescents continues to rise. Vaping occurs when an e-cigarette or vape pen heats a liquid and produces an aerosol that is then inhaled. Excessive use of such devices may lead to inflammation of the lungs, also known as e-cigarette or vaping-use-associated lung injury (EVALI). We present an unusual case of pneumomediastinum and subcutaneous emphysema in a 27-year-old male in the setting of EVALI. Clinicians should be aware of this phenomenon when evaluating patients with a prolonged history of vaping.

## Introduction

Electronic cigarettes (e-cigarettes) were introduced to the US in 2006 and were widely advertised as a smoking-cessation aid, leading many people to perceive them as a safer substitute for tobacco [1]. Manufacturers of vaping products launched aggressive marketing campaigns targeting youth, leading to the popularity of pod-based products due to their design, ease of use, and appealing flavors [2]. On December 18th, 2018, the US Surgeon General officially declared a youth e-cigarette epidemic, followed by the establishment of e-cigarette or vaping-use-associated lung injury (EVALI) by the US Centers for Disease Control and Prevention (CDC) in 2019 [3,4].

The initial EVALI reports described this pathological process as most common in young adult males with a previous history of asthma, cardiac disease, mental health conditions, and obesity. Commonly observed symptoms include dyspnea, cough, chest pain, nausea, vomiting, fever, and weight loss, with typical chest computed tomography (CT) findings as bilateral ground glass opacities [5,6]. While diffuse lung injury is a defining feature of this disease state, delays in diagnosis or treatment can allow complications to extend beyond the pulmonary system. Mediastinitis is becoming increasingly recognized as one such complication. Specifically, spontaneous pneumomediastinum is thought to occur by forced inhalation due to vaping, leading to an increase in intrathoracic pressure, causing alveolar rupture [7]. In addition, severe inflammation injury and pulmonary infections may also predispose patients to the development of mediastinitis [8]. However, the evidence reviewing the progression of EVALI-related complications is still lacking. Herein, we report a case of vaping-associated pneumomediastinum.

## Case presentation

**Figure 1:**
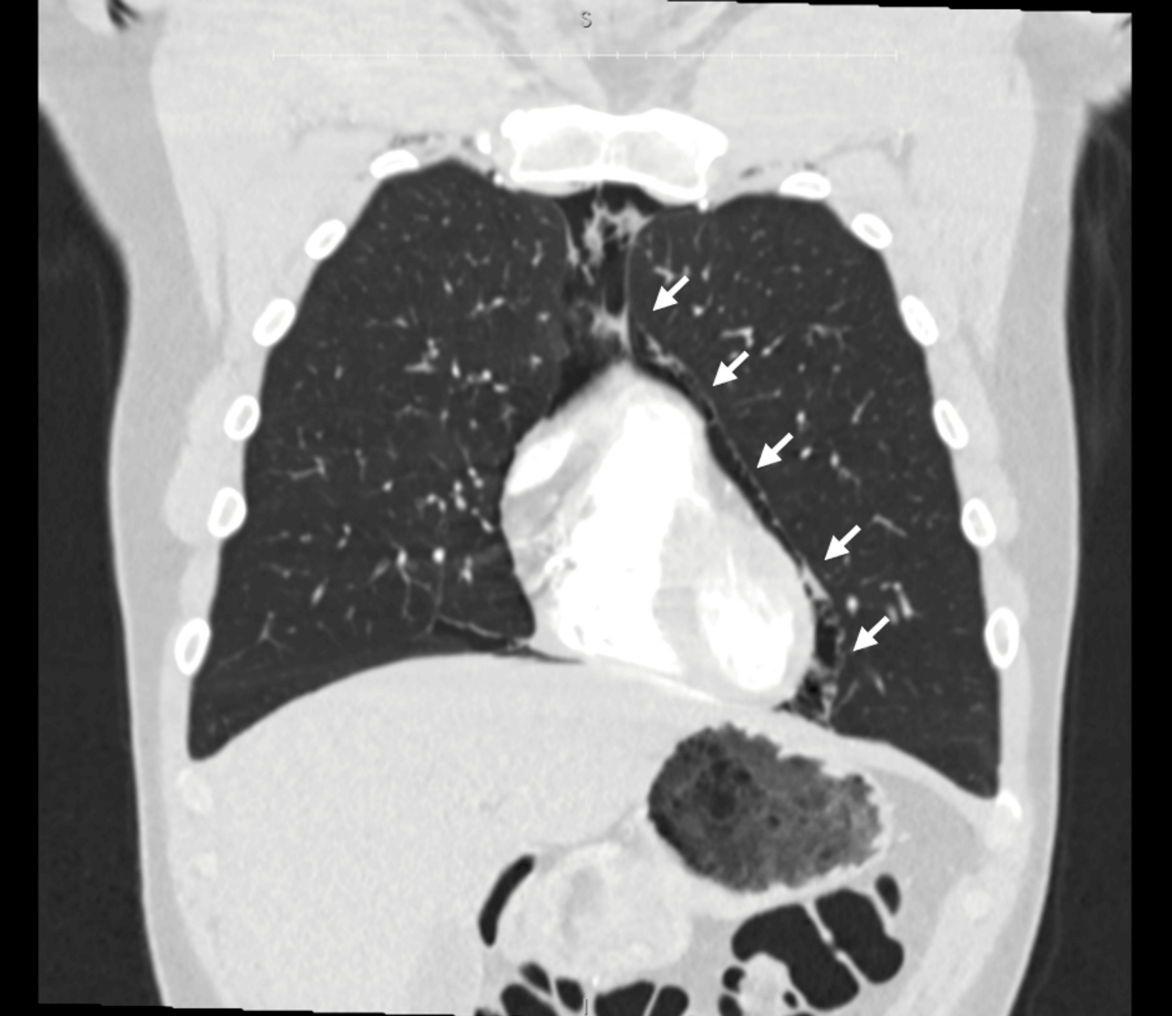
Computed tomography of chest showing pneumomediastinum

A 27-year-old male presented to the emergency room with increased shortness of breath, wheezing, and a productive cough for the past two days. The patient’s past medical history was significant for asthma, anxiety, hypertension, and erectile dysfunction. His social history included vaping and marijuana use. On admission, the patient had a heart rate of 129 beats per minute, a respiratory rate of 22 breaths per minute, an SpO2 of 97%, a body mass index of 26.9 kg/m^2^, and was afebrile. Baseline laboratory findings were within normal limits apart from an elevated white blood cell count, neutrophil count, hemoglobin, and glucose (Table 1). In addition, the patient was negative for influenza A, influenza B, and SARS-COV-2; however, he was positive for the human rhinovirus. The patient was initially treated for asthma exacerbation with azithromycin 500 mg by mouth once, dexamethasone 10 mg intravenous (IV) once, magnesium sulfate 2000 mg IV once, a single dose of normal saline 1000 mL IV bolus, and as-needed nebulized albuterol-ipratropium 0.5 mg-3 mg; however, these provided minimal relief. A chest x-ray was obtained and showed a questionable pneumomediastinum. A follow-up chest CT further confirmed this diagnosis, along with subcutaneous emphysema in the neck and chest wall areas (Figure 1).

**Table 1:** Pertinent laboratory values

Laboratory test (reference values)	Result
White blood cells (4.0-11.0 k/µL)	16.6
Neutrophils (1.5-7.0 k/µL)	15.0
Hemoglobin (13.0-17.0 g/dL)	17.3
Platelets (140-416 k/µL)	265
Sodium (136-145 mmol/L)	142
Potassium (3.5-5.0 mmol/L)	4.3
Chloride (98-107 mmol/L)	104
Bicarbonate (22-31 mmol/L)	25
Glucose (70-100 mg/dL)	107
Blood urea nitrogen (7-26 mg/dL)	14
Creatinine (0.6-1.3 mg/dL)	1.1
Albumin (3.7-4.9 g/dL)	4.5
Alkaline phosphatase (40-150 U/L)	53
Aspartate aminotransferase (15-48 U/L)	25
Alanine aminotransferase (8-56 U/L)	21
Total bilirubin (0.2-1.0 mg/dL)	0.5

The patient was transferred to the medical intensive care unit, where he was placed on 40 mg IV methylprednisolone every 12 hours, 0.4 mcg/kg/hr of IV dexmedetomidine, nebulized budesonide 0.5 mg twice daily, nebulized albuterol-ipratropium 3 mL every four hours, and ketamine continuous infusion running at 0-40 mcg/kg/min. The patient, in turn, became combative and aggressive and began to hallucinate, which is thought to be associated with the ketamine infusion. As a result, the ketamine infusion was stopped, and the dexmedetomidine infusion was adjusted to 1.4 mcg/kg/hr. In addition to the listed medications, 80/20 heliox was initiated to maintain oxygen saturation above 90%. The next day, he was weaned off heliox to an initial high-flow nasal cannula (HFNC) setting of 30 L/min flow and 35% FiO2 that was increased to a setting of 40 L/min flow and 45% FiO2 to maintain a goal oxygen saturation above 92%.

The next day, the patient was reportedly feeling much better, with wheezing markedly improved. He was transitioned to a 5 L nasal canula and soon thereafter to room air. The dexmedetomidine infusion was discontinued, and IV methylprednisolone was transitioned to prednisone 40 mg by mouth once, followed by a taper at discharge. The patient was counseled on complete cessation of vaping in addition to any tobacco or marijuana use.

## Discussion

This case adds to the growing evidence of severe pulmonary complications linked to vaping and highlights the ongoing public health implications of rising e-cigarette use. Vaping-induced pneumomediastinum is a rare complication associated with vape use. One proposed mechanism is by direct lung injury by toxic chemicals [9]. This study showcased diacetyl, nicotine, chlorine, and vitamin E as possible agents. These compounds are commonly found in many liquids within e-cigarettes, and while the exact mechanism is still not fully understood, one possible correlation is due to direct damage to the respiratory epithelium [10]. Flavoring compounds found in these e-cigarettes are also compounding factors in the development of this lung injury. Diacetyl, 2,3-pentanedione, and acetoin are flavoring agents and are attributed to bronchiolitis obliterans. One study found that in 47 of 51 flavors tested, diacetyl was the primary compound in 39 of the 51 flavors [9].

Diagnostic imaging is prudent for patient evaluation and has a characteristic pattern on CT imaging. A retrospective cohort study noted common image findings amongst 160 EVALI patients [11]. Centrilobular nodules, interlobular septal thickening, pleural effusion, and lymphadenopathy were defined in their study as common findings affecting anywhere from 22 to 63% of patients. A different retrospective study found that most of their patient population had diffuse ground-glass opacities noted on CT affecting most or even all the lobes bilaterally [12]. 

Another study looked at how e-cigarettes affected lung physiology [13]. The study found that even a five-minute exposure increased airway flow resistance measured by an oscillometer. In addition, they found that e-cigarettes with and without nicotine both decreased the vital capacity of users after 30 minutes of use. Both human and animal studies showed increased airway resistance, reactivity, and obstruction/inflammation along with emphysema from the use of e-cigarettes [13]. 

Pneumomediastinum is typically self-limiting [14]. However, there are a few causative factors that can exacerbate this condition, and they include: asthma, exercise that requires straining, diabetic ketoacidosis, childbirth, cough/vomiting, and drug inhalation [15,16]. In our case, treatment of the patient's asthma with oxygenation and corticosteroids led to resolution.

## Conclusions

In conclusion, e-cigarettes or vaping can cause significant lung injuries, such as pneumomediastinum, which can lead to hospitalization. To prevent this from happening, healthcare providers are encouraged to screen and counsel patients on the cessation of vaping in addition to any tobacco or marijuana use.
